# QUALITATIVE STUDY IDENTIFIES DEMENTIA TRAINING NEEDS OF CARE WORKERS IN ASSISTED LIVING AND PERSONAL CARE AGENCIES

**DOI:** 10.1093/geroni/igae098.1300

**Published:** 2024-12-31

**Authors:** Troy Andersen, Daniel Kaplan, Norman Foster, Jeanine Stefanucci, Chad Moffitt, Andrea Dremann, Bonnie Bechek

**Affiliations:** University of Utah, Salt Lake City, Utah, United States; Adelphi University, Garden City, New York, United States; University of Utah, Salt Lake City, Utah, United States; University of Utah, Salt Lake City, Utah, United States; University of Utah, Salt Lake City, Utah, United States; ProActive Memory Services, Inc., Salt Lake City, Utah, United States; ProActive Memory Services, Inc., Salt Lake City, Utah, United States

## Abstract

Caring for individuals with dementia can be overwhelming, especially when direct care workers lack knowledge and skills to respond to changing needs of those with dementia. Burnout and turnover are common problems. To better understand wishes for dementia training, we interviewed direct care workers, supervisors and administrators in 4 assisted living facilities (ALFs) and personal care agencies (PCAs). Funded by the NIA, we collected data from 23 direct care workers, 7 supervisors, 8 administrators. Questions focused on needs for dementia training topics and solutions for delivering content. Interviews were recorded, transcribed, and evaluated by two independent raters to identify all topics discussed. These topics were then further analyzed by a second group of raters who specialize in training of direct care providers. Commonalities and thematic differences across direct care workers, supervisors, and administrators were reviewed qualitatively to identify highest priorities. For both ALFs and PCAs, priority topics for training overlapped between administrators, supervisors, and direct care workers and included skill development (e.g., de-escalation strategies), knowledge areas (e.g., bathing, distress), and workforce goals (e.g., training certifications). Direct care workers further mentioned that training should be interactive, fit into their work schedules, and give solutions in real time. PCAs added that greater effort is needed to assure that training is relevant (e.g., current topics often are too pedantic and not practical to daily activities). Real-time, flexible, content meeting identified needs, and individualized to caregiving settings, learner knowledge and experience should be the focus of future improvements in dementia training.

